# Nucleic-Acid-Based Strategies and Nanotechnology Applications for Targeted Therapy

**DOI:** 10.3390/pharmaceutics18020256

**Published:** 2026-02-19

**Authors:** Andrea Patrizia Falanga, Stefano D’Errico

**Affiliations:** Department of Pharmacy, University of Naples Federico II, via D. Montesano, 49, 80131 Naples, Italy; stefano.derrico@unina.it

## 1. Introduction

Since the mid-2010s, the use of nucleic acids as drugs as received considerable attention [1,2,3]. Oligonucleotides, which are short polymers of nucleic acids, show substantial promise for treating and managing a wide variety of diseases. Therapeutic development in this field has largely concentrated on gene silencing [4,5]. However, approaches such as splice modulation and gene activation are also being explored [6,7], extending the range of potential targets beyond those of conventional pharmaceuticals. Most oligonucleotide-based strategies interact with their intended targets through complementary Watson–Crick base pairing, enabling the relatively direct evaluation of candidate sequences [8,9]. In many cases, highly specific lead molecules can be rationally designed using only the primary gene sequence information, with lead candidates identified through rapid screening. Another advantage of oligonucleotide therapeutics is their potential to contribute to precision or personalized medicine because these therapeutics can, in principle, be engineered to selectively target almost any gene with minimal—or at least predictable—off-target effects [5,10]. Oligonucleotide therapeutics may also be designed to act on patient-specific genetic sequences that underlie rare diseases.

In addition to targeting specific sequences through complementary base pairing, nucleic acids interact with proteins through adopting three-dimensional secondary structures, a characteristic under exploration for therapeutic applications [11,12]. The structure of nucleic acids are shaped by intracellular factors, such as ion concentrations and DNA-binding proteins: sequence information that can be applied to stabilize certain secondary forms. Purine- or pyrimidine-rich regions of DNA can form four-stranded arrangements, referred to G-quadruplexes, depending on the nucleotide composition [13]. These structures may occur in functionally important genomic sites, such as telomeres and the regulatory regions of oncogenes [14]. Designing and synthesizing small molecules, such as ligands that can specifically target these unusual DNA structures, and studying their biochemical and biological consequences may inform the development of valuable strategies for cancer therapy [15].

## 2. Overview of Published Work

The purpose of this Special Issue was to collect papers focused on, but not limited to, oligonucleotide synthesis, the biochemical and biophysical characterization of nucleic acids, new oligonucleotide-based delivery systems, as well as biomaterial and polymer development. In the first paper, the authors describe the ongoing exploration of small-molecule ligands that selectively bind to and stabilize G-quadruplex nucleic acid structures. This is a promising avenue in targeted anticancer research given the crucial functions these structures play in regulating cancer cell pathways, as described by Criscuolo et al. [1]. Falanga et al. summarized the existing knowledge on the functions and biological roles of several G4 structures in plants. Some the most promising G4 ligands isolated from plants are potential lead compounds in pharmaceutical field [2]. The delivery of antimiRNA PNAs and microRNA mimics using a series of cationic calix arenes [4] with ammonium and guanidinium moieties is reported by Gasparello et al. [3]. Goga and Stoffel used a polycationic beta-cyclodextrin carrier for nucleoside and nucleotide drugs [4]. In the fifth paper, Sun et al. demonstrate that targeting liver Xor with GalNAc-siRNA is an effective strategy for hyperuricemia therapy, reducing plasma uric acid levels and kidney damage [5]. In the sixth paper, Fedorovskiy et al. discuss the mechanisms of the lipid-mediated cellular uptake of nucleic acids [6]. In the seventh study, an aptamer-drug therapeutic was developed based on EGFR-specific aptamer GR-20 as the targeting element and alkyne-Val-Cit-PABC-MMAE as the payload linker [7]. In the eighth paper, Bauer and Dmitrienko provide an overview of the current state of the art in the use of chemically modified nucleic acid derivatives for therapeutic applications, with a particular focus on oligonucleotides conjugated to lipid moieties. They systematically analyzed the self-assembly ability of amphiphilic oligonucleotides into micelle-like structures as well as the influence of noncovalent interactions between these derivatives and serum albumin on biodistribution and therapeutic outcomes [8]. Recent advances in genetics and nucleic acid chemistry have enabled the creation of fundamentally new tools for practical applications in therapy and diagnostics and for fundamental genome editing tasks. Nucleic-acid-based therapeutic agents offer a distinct advantage of selectively targeting the underlying cause of the disease. Nevertheless, despite the success achieved, unresolved issues remain regarding improving the pharmacokinetic properties of therapeutic nucleic acids while preserving their biological activity. To address these challenges, focus is growing on studying safe and effective delivery methods using modified nucleic acid analogues and their lipid bioconjugates. Bege et al. discussed the relevance of pegaptanib as an aptamer targeting VEGF in diseases such as age-related macular degeneration, where pegaptanib acts as an anti-VEGF molecule [9]. In the final paper, Tufeu et al. comprehensively analyzed the delivery approaches and platforms for RNA-based therapeutics, including ASOs, siRNAs, mRNAs, and aptamers, covering approved therapies and those in late-stage clinical trials [10]. 

This Special Issue highlights the continuous progress and growing diversity of research in the field of nucleic-acid-based therapeutics. This collection of papers illustrates the multifaceted potential of oligonucleotides and related molecules ranging from the exploration of G-quadruplex structures and aptamer design to the development of innovative delivery systems and chemical modifications aimed at increasing stability and specificity, as well as improving pharmacokinetic properties. These studies demonstrate that nucleic acids can serve as not only genetic information carriers but also versatile therapeutic agents capable of highly accurately modulating biological pathways. Advances in the synthesis of modified oligonucleotides, the rational design of aptamer–drug conjugates, and the optimization of lipid- and polymer-based delivery systems have markedly expanded the range of possible clinical applications. Overall, the contributions in this Special Issue emphasize the importance of integrating chemical innovation, structural understanding, and biological validation to overcome current challenges in this field. Continued interdisciplinary efforts are needed to accelerate the translation of nucleic-acid-based therapeutics from the laboratory to clinical practice, paving the way for the development of more effective, targeted, and personalized treatments for a broad spectrum of diseases. Finally, special thanks to all the reviewers, who helped the Guest Editor with clear and final decisions, maintaining the high-quality standards of *Pharmaceutics*.

## 3. Future Perspectives

The Guest Editors think that this field will play a pivotal role in driving future advancements. The steady progress in well-defined research domains, as reflected by the contributions to this Special Issue, highlights a growing interest in the individualization of therapies based on oligonucleotides, which show the potential to profoundly influence the long-term development of the field. As this field continues to grow, these innovative tools may shape the future of nucleic-acid-based therapeutics.
